# Psychological Effects of Hands-On Training Using Public Transportation among Inpatients with Physical Disabilities: Analysis of the Self-Efficacy and Perception of Occupational Enablement Using a Multimethod Design

**DOI:** 10.1155/2020/1621595

**Published:** 2020-12-30

**Authors:** Masahiro Ogawa, Yoriko Hayashi, Tatsunori Sawada, Mizuki Kobashi, Hitoshi Tanimukai

**Affiliations:** ^1^Faculty of Rehabilitation, Kobe Gakuin University, Kobe, Japan; ^2^Department of Rehabilitation, IMS Itabashi Rehabilitation Hospital, Tokyo, Japan; ^3^School of Health Sciences, Tokyo University of Technology, Tokyo, Japan; ^4^Department of Rehabilitation, Kyoto Hakuaikai Hospital, Kyoto, Japan; ^5^Department of Human Health Sciences, Graduate School of Medicine, Kyoto University, Kyoto, Japan

## Abstract

**Introduction:**

This study is aimed at understanding how practicing the use of public transportation can affect the self-efficacy and perceptions of occupational enablement among patients with physical disabilities in a recovery rehabilitation hospital.

**Method:**

We recruited 21 inpatients with physical disabilities caused by stroke or orthopedic diseases from a recovery rehabilitation hospital in Japan and used a multimethod design including an intervention study and a follow-up survey. The intervention study utilized a before-after trial and provided hands-on training in the use of public transportation as the intervention. How self-efficacy and perceptions of occupational enablement changed before and after the intervention was measured using the visual analog scale (VAS). The follow-up survey was conducted to investigate whether patients used public transportation postdischarge.

**Results:**

Only differences in the VAS scores regarding self-efficacy were significant between before and after the hands-on training in the use of public transportation, whereas differences regarding the perceptions of occupation enablement were not. Self-efficacy after the intervention was higher than that before the intervention. In the follow-up survey, both VAS scores of the psychological factors were significantly higher in the group that used public transportation postdischarge than in the group that did not.

**Conclusion:**

Providing hands-on training in the use of public transportation for inpatients with physical disabilities increased their self-efficacy, indicating that psychological factors should be evaluated to predict their occupational skill improvement and to verify the outcomes of an occupational therapeutic intervention.

## 1. Introduction

Public transportation helps people living in urban communities engage in extensive interactions across social environments. For example, using trains and buses can enable some people to commute and others to go shopping. Research has shown that not using public transportation can limit outside participation and narrow an individual's life spaces [1–3]. Especially for people with physical disability and older adults, lack of outdoor mobility can lead to isolation, poor health, high mortality, and low quality of life [4–7]. In urban communities, especially in places with well-developed public transportation system, not being able to use public transportation can be a serious social problem for people with physical disability.

Occupational therapists may play an important role in helping people with physical disability use public transportation. This opportunity involves recognizing the importance of assessing not only the physical but also the psychological factors such as self-efficacy that can enhance a client's performance in daily life. A study of poststroke patients reported that they were reluctant to use public transportation, not only due to physical disability but also more often due to lack of confidence, fear of injury, a lack of information about transport services, etc. [8]. Therefore, it could be highly productive for occupational therapists to directly address these concerns. Another issue regarding occupational therapy aimed at improving the use of public transportation is that rehabilitation aiming at improving the outdoor mobility is generally limited in clinical settings, not to mention the use of trains, buses, and airplanes [9]. Persson and Selander [10] found that among 5-year poststroke patients in an urban population, one-fifth had reported problems with outdoor mobility and that they need transport training during rehabilitation to enhance outdoor mobility. However, these efforts were said to be limited by various barriers, including therapists' lack of knowledge and technical skills and issues on their role identity and availability of resources [9, 11]. Therefore, to help determine the merits of providing more resources to address this need, we investigated the productivity of performing rehabilitation on patients receiving hands-on training in the use of public transportation.

Only a few studies have addressed this issue, especially regarding the psychological aspects that may be involved here. A study by Logan et al. [12], using a randomized controlled trial, found that the longitudinal effect of home-based occupational therapy in order to increase outdoor mobility for patients after stroke expanded the instrumental activities of daily living outside. However, evidence regarding the psychological aspects of allowing patients to practice the use of public transportation remains to be limited.

Our previous study found that majority of recovery rehabilitation hospitals in Japan provided only a one-time field practice session regarding the use of public transportation for a typical patient [13]. In addition, a survey on outdoor mobility and transport training for rehabilitation patients in Australia found that these efforts were rare, especially for poststroke patients [9]. From these results, patients with physical dysfunction seem to be having difficulty in materially improving their physical skills, and therefore, improving their ability to use public transportation has focused on the psychological aspects, such as increasing their sense of self-efficacy or decreasing psychological barriers. An evidence suggested that self-efficacy is one of several potential mediators of the effects of physical activity and that rehabilitation to improve these psychological outcomes would be highly productive [14, 15]. Therefore, these psychological efforts are possibly more effective in enabling patients to use public transportation after returning home from hospital, compared with an actual physical practice. However, no study has yet explored the effectiveness of such physical training in improving the important psychological factors related to using public transportation. Therefore, these effects should be clarified, and better ways in practicing the use of outside transportation more effectively should be identified.

Recovery rehabilitation hospitals in Japan provide intensive therapy aimed at helping patients who suffer from a stroke, hip fracture, and spinal compression fracture, among others, and helping them to return home after they have undergone acute treatment. The hospitals that provide intense rehabilitation for patients with medical issues during the recovery phase for approximately 2 weeks to 2–6 months after disease onset are known as Kaifukuki Rehabilitation Wards (Kaifukuki means “recovery phase” in Japanese). Approximately greater than 80% of the patients in these wards suffer from stroke or hip fracture [16]. As a result, these hospitals in Japan admit many patients with disabilities that makes it difficult for them to go outside, and the Kaifukuki organizations consider it a part of their mission to provide intensive and comprehensive rehabilitation so that the patients can return home to live in their respective communities.

In light of this, the present study is aimed at identifying the effects generated by patients with physical disabilities undergoing recovery rehabilitation to receive hands-on training in the use of public transportation, particularly regarding how possible changes in self-efficacy and perceptions of occupational enablement might be affected. To this end, an intervention study and a follow-up survey were used in a recovery rehabilitation hospital.

## 2. Methods

### 2.1. Participants

Inpatients with physical disabilities caused by stroke or orthopedic diseases resulting in limited mobility were recruited from a recovery rehabilitation hospital in Tokyo. All participants were selected according to the following five inclusion criteria: (1) having a hope to use a train or bus after their discharge, (2) score of greater than 24 points on the Mini-Mental State Examination (MMSE) and no apparent symptoms of higher brain dysfunction, (3) scheduled to be discharged to their home, (4) their home located nearer than 1 km from a train station or bus stop, and (5) a score of greater than 6 points on the Walk/Wheelchair section in the motor subscale of the Functional Independence Measure (FIM), demonstrating modified independence. These criteria are aimed at recruiting patients who had the potential to independently use a train or a bus after their discharge.

All patients had been hospitalized between December 2015 and December 2017. The hospital had 150 hospital beds and approximately 600 inpatients per year. In this hospital, the inclusion criteria appeared to apply to 15%–20% of the patients based on estimates from our previous research. Approximately 100 rehabilitation staff members, occupational therapists, and physical therapists were employed in the hospital, and five of these cooperated in this study. These collaborators were informed about participant selection, study procedures, and ethical considerations before the data collection. Participants were recruited into the study after an introduction by the collaborators.

Before data collection, all patients were given written and oral information about the study, including how the results would be used, and informed consent was obtained. This project was approved by the ethics committee at the IMS Itabashi Rehabilitation Hospital (approval number: B010).

### 2.2. Procedures

**Figure 1:**
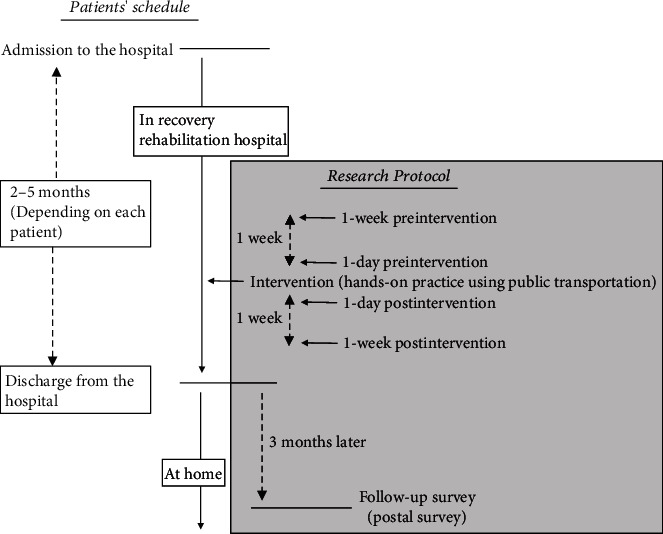
Patients' schedule and the research protocol.

For this investigation, a multimethod design consisting of an intervention study, which was performed while the patients were in the hospital, and a follow-up survey, which was performed after they were discharged, was used (Figure 1).

The intervention study utilized a before-after trial. Each patient was provided hands-on training in the use of public transportation out of the hospital as the intervention. A one-time session, lasting between 1 and 3 h, was implemented where patients were practicing several physical aspects of using a bus or train. The training for each patient was tailored to their individual capabilities and needs. These interventions were performed by a managing occupational or physical therapist of the hospital, or sometimes by both together. Psychological assessments, mentioned in the following section, were performed four times before and after the intervention as follows: 1-day and 1-week preintervention and 1-day and 1-week postintervention.

As part of the standard rehabilitation process, many patients are allowed to practice using public transportation before they are discharged from the hospital. Therefore, the timing of these practice sessions followed the standard rehabilitation process in this study. In this rehabilitation hospital, all patients received nearly 3 hours of the usual rehabilitation program, performed every day.

The follow-up survey was conducted by mail with patients 3 months after discharge from the hospital. Three months after discharge to home, a questionnaire was sent to each patient asking whether they now use public transportation or not.

Considering the clinical ethics, a case-control study could not be performed due to the fact that a control group could not be recruited. Therefore, we selected the before-after trial in accordance with the actual clinical condition. Moreover, adding the follow-up survey was thought to help clarify the effectiveness of the hands-on training.

### 2.3. Psychological Assessments in the Hospital

This study primarily is aimed at exploring if receiving hands-on training in the use of public transportation would produce a positive impact on self-efficacy and perceptions of occupational enablement. Changes in these values were measured using the visual analog scale (VAS), which is easy to use and has been widely employed to assess pain in several healthcare contexts [17, 18]. The VAS is based on a continuous scale, comprising a 10 cm horizontal line anchored at either end by two verbal descriptors, for example, from “no pain” (score of 0) to “unendurable pain” (score of 100). In this study, the same method was used for the following two assessments.

Self-efficacy is a task-specific predictor of behavior and defined as a person's belief in his or her capability to execute an action required to produce a particular outcome [19]. VAS has been used to measure self-efficacy in standardized specific tasks [20]. For this study, the scale was modified to address self-efficacy of using public transportation. Therefore, we asked our patients the following question: “Indicate how confident you are to use a train (or a bus) by yourself after discharge.” The two anchoring verbal descriptors were “Not at all confident” and “Extremely confident.” A higher score indicates higher self-efficacy (test–retest reliability, *r* = 0.96).

Perceptions of occupational enablement were a part of the Assessment of Client's Enablement instrument. This assessment was originally developed to determine a gap between the therapists' understanding and that of the patient regarding occupational performance in daily life, using VAS [21]. In this study, perceptions of occupational enablement were defined as a person's view of how they can execute an occupation in his or her daily life after discharge. By referring to the prior study [21], VAS was used to assess perceptions of occupational enablement regarding the use of public transportation. The question was, “A person's view of how able are you to use a train (or a bus) in your daily life after your discharge?” The two anchoring verbal descriptors were “Do not use in daily life” and “Use in daily life.” A higher score represents higher perceptions of occupation enablement (test–retest reliability, *r* = 0.96).

### 2.4. Assessments of Functions and Abilities before Their Discharge

Variations in motor function, cognitive function, and activities of daily living (ADL) all had the possibility of affecting whether or not our patients chose to use public transportation. To help control these possible influences, several assessments regarding motor function and ADL performance were performed before our patients were discharged from the hospital. The Berg Balance Scale (BBS) [22] and the 10-meter Walking Test (10mWT) [23] were used as indices of motor function. Likewise, MMSE [24] and FIM [25] were also used to evaluate the cognitive function and ADL abilities.

### 2.5. Questionnaires in the Follow-Up Survey

In the follow-up survey, a postal survey was used to ask patients if they had used public transportation or not. To examine the effect of using public transportation, several additional questionnaires were also used. These included an 8-Item Short-From Health Survey (SF-8) [26], a Life Space Assessment [27], and the Index of Competence (TMIG-IC) developed by the Tokyo Metropolitan Institute of Gerontology [28]. We regarded these three instruments as indices of the quality of life, life space, and instrument ADL, respectively. A higher score on these questionnaires indicates higher quality of life and ability. All of them were self-administered by our patients at their homes, 3 months after discharge from the hospital.

### 2.6. Statistical Analysis

To evaluate the psychological effects that might have been generated by our patients who underwent training to use public transportation, VAS scores addressing self-efficacy and perceptions of occupation enablement with regard to using public transportation were compared across the following four time periods: 1-week preintervention, 1-day preintervention, 1-day postintervention, and 1-week postintervention. To compare the data among these four periods, the Friedman test was performed, followed by the Wilcoxon signed-rank test with Bonferroni adjustment as a post hoc test.

We also compared patients according to their use of public transportation. Patients were divided into the two following groups: those who used public transportation after discharge (use group) and those who did not use it (nonuse group). For this comparison, a two-sided Mann–Whitney *U* test was used. These two variables analyzed were VAS scores of self-efficacy and perceptions of occupation enablement. Moreover, the two-group comparison was also performed for categories according to patients' age, BBS, 10mWT, MMSE, and FIM in the hospital. In addition, the SF-8, Life Space Assessment, and TMIG-IC were also compared between the two groups.

All analyses were performed using IBM SPSS Statistics 24. A *p* value of <0.05 was considered significant.

## 3. Results

### 3.1. Participants

A total of 21 patients (mean age, 67.4 years; SD, 11.4; female, 14) were recruited for this study. Their mean hospitalization period was 93.2 (SD: 45.0) days. Their primary diagnosis for hospitalization was central nervous system disease, such as stroke (13 patients) and orthopedic disease of the femur or spine (8 patients). The mean (SD) score on assessing functions and abilities before their discharge was 48.0 points (8.1) as measured by the BBS, 10.2 s (6.4) per 10mWT, 29.0 points (1.5) on the MMSE, 107.1 points (36.5) on all FIM items, 84.3 points (76.2) on the motor FIM items, and 34.1 points (2.3) on cognitive FIM items.

### 3.2. Intervention Study

In this aspect of the investigation, the practice session was executed at an average of 18.9 (SD: 16.2) days before the patients' discharge. Data regarding the 1-week preintervention were missed in one patient.

**Figure 2:**
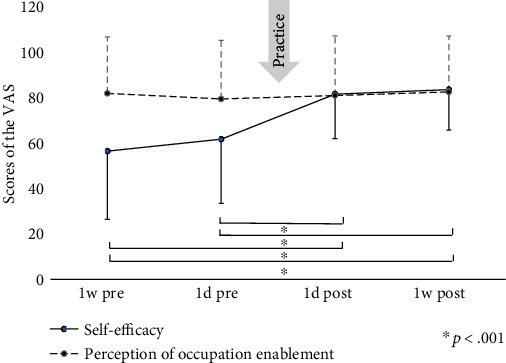
Changes in the visual analogue scale scores on self-efficacy and perceptions of occupation enablement at four periods before and after the practice. 1 w pre: 1-week preintervention; 1 d pre: 1-day preintervention; 1 d post: 1-day postintervention; 1 w post: 1-week postintervention.

Changes in VAS scores of self-efficacy and perceptions of occupation enablement at four periods before and after the practice session are shown in Figure 2. Means (SD) of the self-efficacy scores were 56.4 points (30.0) on the 1-week preintervention, 61.7 points (28.2) on the 1-day preintervention, 81.5 points (19.5) on the 1-day postintervention, and 83.5 points (17.8) on the 1-day postintervention. Means (SD) of perceptions of occupation enablement score were 81.8 points (24.9) on the 1-week preintervention, 79.4 points (25.8) on the 1-day preintervention, 80.9 points (26.2) on the 1-day postintervention, and 82.5 points (24.5) on the 1-day postintervention.

During these four periods, a significant change in VAS scores regarding self-efficacy was observed (Friedman test, *p* < 0.001). Post hoc analysis showed that differences in four comparisons of self-efficacy were significant as follows: 1-week preintervention vs. 1-day postintervention (*p* < 0.001), 1-week preintervention vs. 1-week postintervention (*p* < 0.001), 1-day preintervention vs. 1-day postintervention (*p* < 0.001), and 1-day preintervention vs. 1 week postintervention (*p* < 0.001). Except for these cases, no significant differences were observed in the post hoc test. In other words, significant differences were only observed regarding self-efficacy during the entire practice.

No significant differences were observed on VAS scores regarding the perceptions of occupation enablement (*p* = 0.26). To analyze the cause of this finding, changes between before and after practice sessions were examined.  Table 1 presents the stratified distribution of VAS score changes. The self-efficacy scores for majority of patients were increased after the practice experience. In contrast, four patients had lowered their perceptions of occupational enablement.

### 3.3. Follow-Up Study

Five patients did not use either the train or bus after discharge and were classified into the nonuse group, whereas the remaining 16 patients used either the train or bus and were classified into the use group.

Comparisons of assessments between the use and nonuse groups are shown in Table 2. Significant differences on self-efficacy, perceptions of occupational enablement, BBS, 10mWT, all FIM items of the cognitive domain, and TMIG-IC were observed. These results indicate that both psychological and physical factors, as well as the ADL ability in the use group before their discharge, were significantly better than those of the nonuse group. Furthermore, the independence of instrument ADL ability in the use group was considerably higher than that in the nonuse group.

## 4. Discussion

This study found that providing inpatients with physical disabilities with hands-on training in the use of public transportation increased their self-efficacy in a recovery rehabilitation hospital. This result suggests that assessments of psychological factors, such as self-efficacy, could be important indices in examining the effectiveness of such training efforts. Lack of confidence was reported as one of the barriers in using public transportation for patients with physical abilities caused by stroke [8]. Our results show that a patient's self-efficacy toward the use of public transportation should be assessed and improved to remove this barrier in the use of public transportation. One way to do this is to provide patients with successful experiences [19]. In this context, enabling patients to practice and actually use public transportation may be a cost-effective approach to providing a successful and empowering experience.

Conversely, VAS scores of our patients were found to decline regarding the perceptions of occupational engagement after the practice sessions. In other words, the practice reduced their motivation to use public transportation after their discharge from the hospital. This clearly resulted from an adverse reaction to the practice experience, which may have generated a sense of shame in connection with venturing outside through the public transportation. Therefore, future studies should examine in detail the reasons for patients' perceptions of decreased occupational engagement.

Assessments of psychological factors such as self-efficacy and perceptions of occupational engagement are frequently overlooked in clinical settings. After discharge, psychological assessment results of the nonuse group were significantly lower than those in the use group. Thus, these assessments appear to be highly valuable in determining a patient's ability to undertake independent activities. Therefore, evaluating these psychological factors may help predict whether enabling occupation has been achieved and to verify an outcome of occupational therapy.

However, not only the psychological factors but also the physical abilities involved, such as balance and walking, should be evaluated. In this study, the use group had significantly higher physical abilities than the nonuse group. Our previous follow-up study after discharge from a rehabilitation hospital indicated that the BBS score in the hospital was one of most essential factors in predicting how the patient with physical disabilities would evaluate the pros and cons of using public transportation after discharge [29]. Regression analyses in this previous study found that a cut-off point on the instrument was 43 points for using trains and 45 points for using buses. The mean BBS in the nonuse group was 39.2 points, i.e., below the cut-off point. Therefore, physical capabilities represented by the ability to maintain a good balance, as well as the psychological factors involved, should be improved and assessed before engaging in practice sessions to use public transportation because improving balance ability through a one-time practice only is challenging.

Allowing patients with physical abilities to use public transportation could materially enhance their independence and general social life after discharge. We found that a higher TMIG-IC indicates higher social independence, social activity, and social role [28], all of which are known to be highly productive in supporting their general mental and physical health. In other words, using public transportation enables people with physical disabilities to perform a much wider range of activities. Logan et al. [12] reported that an occupational therapy intervention increased the outdoor mobility after stroke as determined by a randomized controlled trial. Our results support that of a previous research and specifically demonstrate the significant contribution of occupational therapy toward increasing outdoor mobility and community life of patients with physical disabilities under rehabilitation, in particular by increasing their use of public transportation.

## 5. Limitations and Future Research

The small number of patients in this study limits the generalizability of its findings, especially during the follow-up period. Considering the clinical ethics involved, allocating patients to the control and intervention groups was impossible; hence, the intervention study and the follow-up survey were conducted. Consequently, the ratio of the nonuse group in the follow-up study was low, and the number of observations was limited. Moreover, this study only involved one facility in Japan. In future research, this subject should be investigated using multiple facilities located in diverse regions.

In this study, including mixed diagnostic categories within the sample contaminated the findings. It would have been better to focus only on patients with stroke or other disease.

Various psychological factors examined in this study were not directly explored further after discharge regarding their impact on the use of public transportation because several physical factors related to this decision can generate many confounding factors, making it difficult to isolate the psychological elements. Another limitation was the design of the before-after trial, which was unable to provide data that would allow us to establish firm conclusions regarding the intervention. Therefore, in the future, our research on the relationship between psychological and physical factors involved will be analyzed in a much greater depth. Such forthcoming investigations may generate insights as to how we can better prepare patients with physical disabilities under rehabilitation to use public transportation.

## 6. Conclusion

This study found that giving patients with physical disabilities hands-on training in the use of public transportation increased their sense of self-efficacy, which may have improved their willingness to use public transportation after discharge, in a recovery rehabilitation hospital practical. These findings support the idea that allowing patients to practice using public transportation can be highly productive. Our results also indicate that evaluating psychological factors involved is important because they may predict the factors that can lead to enabling occupation and help verify an outcome of an occupational therapy.

### 6.1. Key Findings

The following are the key findings:

- A practice of using public transportation for patients with physical disabilities increased the self-efficacy
- The self-efficacy might affect the use of public transportation in their actual activities of daily living

### 6.2. What the Study Has Added

This study suggests that psychological factor assessments, such as self-efficacy, could be important indices to use in examining the effectiveness of training patients with physical disabilities in using public transportation. Moreover, the results of this study highlight that hands-on training in the use of public transportation is a meaningful and relevant rehabilitation method to increase the patients' self-perceived efficacy and to facilitate the use of public transportation.

## Figures and Tables

**Table 1:** Stratified areas of distribution on the change amount of visual analogue scale (VAS) scores (unit: number of patients).

Range of change amount	SE	POE
Over +19	11	4
+10 to +19	4	1
0 to +9	6	12
−10 to −1	0	1
−20 to −11	0	1
Under −20	0	2

SE: self-efficacy; POE: perceptions of occupation enablement. The change amount was subtracted from the VAS score of 1-day preintervention from that of 1-day postintervention. The table shows the number of patients according to different change amount categories.

**Table 2:** Comparisons of assessments between the use and nonuse groups.

Assessments	Use group (*n* = 16)		Nonuse group (*n* = 5)		*p* value	Effect size
	Mean	SD	Mean	SD		*η*_2_
Age (years)	66.1	12.0	73.8	9.2	.066	.131
In the hospital before discharge						
Self-efficacy	89.5	13.3	64.2	17.4	.011^∗^	.347
Perception of occupational enablement	90.8	17.6	55.8	20.2	.006^∗^	.379
BBS	50.8	4.5	39.2	11.3	.025^∗^	.254
10mWT (seconds)	8.5	3.0	15.6	11.1	.040^∗^	.205
MMSE	29.1	1.4	28.6	1.7	.553	.026
FIM	120.4	7.4	110.0	7.7	.049^∗^	.215
Motor	85.8	7.0	79.0	6.4	.077	.175
Cognitive	34.6	1.5	31.4	3.8	.041^∗^	.233
Follow-up study after discharge						
SF-8	18.9	7.5	25.4	10.2	.179	.099
LSA	70.0	29.6	53.8	23.4	.395	.092
TMIG-IC	11.6	1.7	7.8	2.0	.004^∗^	.277

BBS: Berg Balance Scale; 10mWT: 10-meter Walking Test; MMSE: Mini-Mental State Examination; FIM: Functional Independence Measure; SF-8: 8-Item Short-From Health Survey; LSA: Life Space Assessment; TMIG-IC: Tokyo Metropolitan Institute of Gerontology Index of Competence. In this comparison, VAS scores on the 1-week postintervention of self-efficacy and perceptions of occupational enablement were used. ^∗^*p* < .05.

## Data Availability

The raw and processed data required to support the findings of this study cannot be shared at this time due to ethical reasons.
